# Neonatal status epilepticus: critical issues in clinical practice

**DOI:** 10.3389/fneur.2026.1855017

**Published:** 2026-07-16

**Authors:** Elena Pavlidis, Paola De Liso, Gaetano Cantalupo, Elisabetta Amadori, Luca Bartolini, Anna Cavalli, Robertino Dilena, Valentina Gentile, Massimo Mastrangelo, Francesco Pisani, Jacopo Proietti, Elisabetta Cesaroni, The Neonatal Seizures and Epilepsy Committee of the Italian League Against Epilepsy, Elisabetta Cesaroni, Paola De Liso, Gaetano Cantalupo, Elisabetta Amadori, Luca Bartolini, Anna Cavalli, Lucrezia De Cosmo, Robertino Dilena, Valentina Gentile, Silvia Lori, Massimo Mastrangelo, Elena Pavlidis, Francesco Pisani, Jacopo Proietti, Federico Raviglione

**Affiliations:** 1 Child Neurology and Neurorehabilitation Unit, Department of Pediatrics, Regional Hospital of Bolzano (SABES-ASDAA), Teaching Hospital of Paracelsus Medical University (PMU), Bolzano, Italy; 2 Neurology, Epilepsy and Movements Disorders Unit, Bambino Gesù Children's Hospital, IRCCS Full Member of European Reference Network EpiCARE, Rome, Italy; 3 Innovation Biomedicine Section, Department of Engineering for Innovation Medicine, University of Verona, Verona, Italy; 4 Child Neuropsychiatry Unit, University Hospital of Verona (full member of the European Reference Network EpiCARE), Verona, Italy; 5 Center for Research on Epilepsy in Pediartic Age (CREP), University Hospital of Verona, Verona, Italy; 6 Department of Clinical Pediatrics Sciences, Unit of Child Neuropsychiatry, Member of the ERN Epicare Network, IRCCS Istituto Giannina Gaslini, Genoa, Italy; 7 Department of Neuroscience, Meyer Children's Hospital IRCCS, Florence, Italy; 8 Department of Neuroscience, Psychology, Pharmacology and Children's Health (NEUROFARBA), University of Florence, Florence, Italy; 9 Neurophysiopathology Unit, Fondazione IRCCS Ca' Granda Ospedale Maggiore Policlinico, Milan, Italy; 10 IRCCS Istituto delle Scienze Neurologiche di Bologna, U.O.C. Neuropsichiatria dell'età pediatrica, Bologna, Italy; 11 Department of Pediatric Intensive Care Unit, IRCCS Policlinico San Donato, Milan, Italy; 12 Department of Human Neuroscience, Sapienza University of Rome, Rome, Italy; 13 Child Neurology and Psychiatric Unit, Pediatric Hospital G. Salesi, Azienda Ospedaliero-Universitaria delle Marche, Ancona, Italy

**Keywords:** neonatal neurology, neonatal seizures, neonatal status epilepticus, neuromonitoring, status epilepticus

## 1 Introduction

Neonatal status epilepticus (NSE) is a long-standing controversy and, at the same time, a current topic of active debate. The International League Against Epilepsy (ILAE) definition of status epilepticus, based on operational dimensions T1 (time after which a seizure is likely to be prolonged) and T2 (time after which long-term consequences may occur), represents a cornerstone for clinical management in older children and adults (1–5). However, data concerning the possibility of translating the same approach to the neonatal period are still missing. Neonates are excluded from this definition due to maturational peculiarities, and to developmental differences in seizure mechanisms and manifestations. In the neonatal period seizures are often electrographic-only, sometimes brief but often recurrent. Access to continuous video-EEG-monitoring, essential for seizure diagnosis and characterization (6), varies widely across centers, with the result of an inadequate analysis of the electroclinical phenomenon. The lack of a shared operational definition of NSE continues to hinder clinical decision-making and research comparability.

Here, we highlight the still pending conceptual dilemmas and practice uncertainties point-by-point.

## 2 The unique neonatal brain

As previously highlighted in the literature (7), the neonatal brain has distinct electrophysiological and biochemical features. It shows an extremely low threshold to seizures due to an imbalance toward an hyperexcitable state. Immature GABAergic networks may be excitatory rather than inhibitory. During early development, GABA signaling is depolarizing, inducing outward chloride currents due to high intracellular chloride concentrations mediated by the NKCC1 cotransporter. The postnatal shift from depolarizing to hyperpolarizing GABA represents a pivotal event in brain development, and altered timing of this shift is associated with both neurodevelopmental disorders and epilepsy. The excitatory GABA action may contribute to the higher seizure propensity and limited anticonvulsant efficacy observed in neonates. The neurotransmitter system remains incomplete during this period, resulting in the highest incidence of seizures across the lifespan and poor response to commonly used antiseizure medications.

Nonetheless, experimental studies in animal models indicate that the immature brain may be less susceptible to seizure-induced injury than the mature brain. In commonly used models of status epilepticus, hippocampal injury is difficult to induce in rats younger than 21 days (8). This relative resistance to acute morphological damage has been attributed to several protective mechanisms, including reduced calcium influx, lower synaptic density and activity, decreased energy consumption, higher levels of brain-derived neurotrophic factor, and better preservation of GABA synthesis during prolonged seizures (9, 10).

Moreover, incomplete myelination of neonatal brain structures leads to the focal/multifocal and recurrent appearance of neonatal seizures, often with subtle or absent clinical correlates.

Newborns represent a heterogeneous population. Variations in gestational age, weight and perinatal complications have an undeniable impact on the incidence and manifestation of NSE, and influence management and treatment response (11–13). Furthermore, considering premature newborns, early external environment exposure modifies the fetal exposome and leads to both overexposure and deprivation in certain sensory domains, further modifying the developmental trajectory (14).

## 3 Criteria and features for NSE definition

### 3.1 Time/duration

The absence of universally shared temporal references for NSE represents the main limitation in clinical management. The paucity of both preclinical and clinical evidence does not allow the identification of a cut-off for the failure of seizure termination mechanisms or initiation of mechanisms leading to abnormally prolonged seizures, nor a duration beyond which seizure activity may determine neuronal injury and alteration of neuronal networks. One of the key limitations is the difficulty in recreating an animal model of neonatal seizures (15). The core dilemma in NSE is whether adult-derived temporal thresholds (T1/T2) are physiologically meaningful for the neonatal brain. Seizures in neonates tend to be shorter, and often recurrent. Their propensity for spontaneous termination on one side and tendency to recur on the other raise questions about whether a continuous-duration criterion alone can capture the concept of NSE.

The proposed definitions of NSE rely mostly on the temporal criterion. Most of them focus on the duration threshold of a single seizure that should be considered as status, though shared agreement on this cutoff has not been reached (16–25). Some authors suggest adopting total seizure burden—the sum of durations of individual seizures within a given time interval—as a more suitable approach to NSE (26). Numis et al. demonstrated that pretreatment maximal hourly seizure burden is strongly associated with lower likelihood of response to initial antiseizure medication, emphasizing the relevance of cumulative burden rather than isolated duration (27). However, it remains unclear whether, in cases with comparable total amount of seizure time, a prolonged single seizure has a similar or different impact compared to the sum of repeated seizures.

Assessing clinical recovery at the end of a seizure or during the interval between recurring seizures is particularly challenging. Furthermore, while the minimum time criterion that defines NSE onset is debated, no guidelines exist regarding when NSE should be considered concluded. For instance, a minimum seizure-free interval needed after the end of the last seizure has not been established, nor is it clear whether seizure relapse minutes or hours apart should be considered a new epileptic event or a continuation of the same status. This latter aspect is particularly relevant in the context of repetitive seizures and has implications both in research (i.e., reporting the overall duration of NSE, or the number of NSE episodes in a single patient, and addressing prognostic implications), and in real-time therapeutic decision-making.

As no predictors exist yet to identify at an early time point those seizures that will end in a brief time and those that will last longer, some authors studied the relationship between the duration of a seizure and that of the subsequent ones, revealing a positive monotonic relationship between the duration of successive seizures and determining to settle a 5-min threshold (28). No subsequent studies have further investigated this finding, therefore there has been no further validation of this time point.

A further question is raised by paroxysms of rhythmic activity such as brief rhythmic discharges that lie on the ictal-interictal continuum. Studies show that finding them is associated with acute brain injuries and carries prognostic implications (29), but given the lack of evolution in frequency, amplitude, morphology and location they do not really qualify as seizures (30). Whether or not to include them in the definition of status epilepticus remains a subject of active debate (31).

### 3.2 Semiology

Most neonatal seizures have absent or ambiguous clinical correlation and video-EEG confirmation is mandatory for their diagnosis. This is also due to the electroclinical uncoupling: the persistence of electrographic seizures despite suppression of clinical manifestations after antiseizure medication administration, which has been documented in up to 58% of neonates with persistent seizures (32).

The ILAE position paper on classification of neonatal seizures redefined semiology categories based on the predominant clinical manifestation (6). Sufficient evidence indicates that all types of seizures, including purely electrographic seizures, impact neural circuits and long-term outcomes (33). However, whether different seizure semiology types have varying short-term and long-term impacts in the context of status epilepticus remains almost entirely unexplored.

In addition to its clinical manifestations, the characteristics of the epileptic discharge are also highly variable in terms of frequency, amplitude, morphology, localization and spread. Differences in these features, which are also understudied, could imply different consequences on neurological function.

In order to accurately characterize the above-mentioned features and to document the presence or absence of a simultaneous clinical manifestation, the use of continuous multichannel video-EEG monitoring is needed, with simultaneously recorded video and polygraphy (34–36).

### 3.3 Etiology

As the main determinant of outcomes, etiology represents a key element to consider both in clinical practice and in research and therefore a fundamental descriptor of NSE.

A primary distinction must be made between status epilepticus as an expression and consequence of an acute event and therefore limited to the time frame surrounding the event itself, and status epilepticus in the context of neonatal-onset epilepsy.

The clinical context and semiology of seizures often guide the etiology identification (37). Clonic NSE is often the presentation of an acute vascular etiology, while predominantly electrographic seizures occurring during the secondary phase of injury are common in neonatal hypoxic-ischemic encephalopathy (38).

Background EEG activity might help to early disentangle between acute symptomatic seizures and neonatal epilepsies (39, 40).

Sequential or tonic seizures are characteristic of neonatal-onset epilepsy with a genetic etiology. Their timing of onset, frequency, duration and temporal distribution and whether they constitute NSE, provide clues in differentiating self-limiting forms from neonatal onset epileptic encephalopathies (41, 42).

### 3.4 Outcome

Identifying meaningful descriptors of NSE must be guided by their correlation with outcomes. Determining the independent contribution of neonatal seizures to neurological development is complex, as long-term outcomes depend strongly on etiology, with numerous other factors playing variable roles in individual subjects. Nonetheless, emerging evidence indicates a correlation between seizure burden or seizure duration in different etiologies and neurological/neurodevelopmental impairment (43–45), with some authors identifying specific thresholds related to adverse outcomes (44, 46, 47) even after adjusting for the severity of brain injury (44, 47, 48).

Despite the apparent higher resistance of the neonatal brain to acute structural injury, increasing evidence suggests that neonatal seizures may still contribute to adverse neurodevelopmental outcomes through alternative mechanisms (49).

In neonatal HIE, the most accurately studied etiology group, the contribution of prolonged or frequent seizures to poorer outcomes is still evident after controlling for clinical severity, EEG background and MRI scores (50, 51). The evolution of the background after the insult (namely the time to regain continuity and cyclicity) remains however a much more informative prognostic factor when looking at neurodevelopment and epilepsy risk in this population (52, 53).

RCTs comparing treatment of electrographic and electroclinical seizures and treatment of electroclinical seizures only showed an overall lower seizure burden in the group in which both electrographic and electroclinical seizures were treated, and a correlation between increasing seizure burden and cognitive, motor and language outcomes. These findings support the premise that electrographic seizures are as detrimental as electroclinical seizures and should be treated equally (33).

Considering these data, a prompt intervention to terminate seizures has been advocated.

### 3.5 Treatment response

Evidence suggests that timely treatment of neonatal seizures results in better response (54). However, in real-life settings where the clinician primarily involved in the management is often the neonatologist and the accuracy of seizure diagnosis relies on the availability of EEG machines, technicians, and neurophysiology interpretation, the appropriateness and timing of treatment are often critical. The concept of a multidisciplinary neuro-NICU team is emerging as essential for optimal care.

It has also been proposed to consider the response to treatment as part of the concept of NSE (21, 55). Nonetheless, there is no agreement on what constitutes an effective therapeutic response, with different percentages of seizure reduction and different times of seizure freedom being adopted.

Moreover, pharmacological options at our disposal have largely remained the same for decades and the use of most ASMs is off-label in the neonatal period. Evidence-based treatment guidelines still rely on phenobarbital as first-line, but recently a targeted approach in some specific etiologies has been advised (56–58). When a channelopathy is the suspected cause of seizures due to typical electroclinical presentation and known family history, sodium channel blockers such as phenytoin or carbamazepine should be considered as a first-line therapies (57). Although supported only by preliminary evidence, a similar recommendation has recently been suggested for the treatment of acute symptomatic seizures secondary to neonatal arterial ischemic stroke, in which the use of phenytoin appears to be more effective than phenobarbital in inducing seizure termination (59).

Furthermore, pyridoxine or PLP supplementation for suspected vitamin B6-dependent epilepsies represents a mechanism-based treatment strategy. Precision-medicine approaches, increasingly relevant in neonatal epilepsy, highlight the need for rapid diagnostic pathways, that integrate biochemical and genetic screening in refractory or atypical seizure presentations.

A proper assessment of treatment response should consider the appropriateness of the treatment itself, and other factors influencing neonatal seizure presentation, such as sedative use or, in the specific case of hypoxic-ischemic encephalopathy, therapeutic hypothermia.

The relationship between treatment and outcome prognostication in neonatal status epilepticus is fraught with unresolved contradictions. While observational data consistently demonstrate that a higher seizure burden is independently associated with worse cognitive and language outcomes (48), randomized controlled trials have failed to demonstrate that more aggressive EEG-guided seizure treatment improves neurodevelopmental outcomes (60). This paradox is compounded by the limited efficacy of available antiseizure medications (61), as nearly 50% of neonatal seizures are refractory to first-line phenobarbital and an additional 30% do not respond to second-line agents, raising the question of whether treatment failure itself confounds prognostic models or whether refractoriness is merely a marker of the severity of the underlying brain injury.

Until adequately powered multicenter neonatal trials can disentangle the independent contributions of seizure burden, treatment response, medication neurotoxicity and underlying etiology to long-term outcomes, outcome prognostication in neonatal status epilepticus will remain linked to treatment decisions that are themselves based on insufficient evidence.

The challenge of balancing treatment benefits and medication-induced neurotoxicity raises question about when to start treatment and when to stop it and what role the neuroprotective strategies beyond therapeutic hypothermia might have.

### 3.6 Consensus-based guidelines and recommendations and real-world practice

Updated consensus-based recommendations from the ILAE Neonatal Task Force (57) underscore the need for standardized antiseizure medication algorithms, EEG monitoring, and early identification of treatable etiologies. American guidelines provide updated indications for neonatal seizure diagnosis, with conventional EEG monitoring being worldwide-recognized as the gold standard (62, 63).

However, real-world practice often deviates from these standards: long-term EEG monitoring (at least 24–48 h) remains limited to a few highly specialized centers; neonatologists rather than epileptologists manage most cases and treatment decisions are often based on aEEG or on clinical impression alone. These gaps highlight the need for improved access to neurodiagnostics and structured care pathways. The INNESCO consensus proposed a multimodal and multilevel approach in order to provide different options tailored to different clinical settings (64).

Selection bias could represent an additional confounding factor to consider when discussing NSE. In neonates, purely electrographic seizures and seizures with subtle clinical correlate are very frequent. Although monitoring with video-EEG/aEEG only in patients deemed at risk for seizures represents a more affordable (and still difficult) clinical approach, it may result in failure to diagnose non-monitored NICU patients who experience electrographic seizures. Moreover, the timing of monitoring plays an essential role in diagnosis and prognosis, with later diagnosis increasing the risk of outcome implications.

Further progress will depend on the integration of new technologies in support of conventional EEG. Machine learning and deep learning seizure prediction models, as well as seizure detection algorithms now entering the market, will become useful in assisting expert neurophysiologists in the timely recognition and earlier treatment of the seizure events (65–67). Signal analysis software may assist in extracting and characterizing quantitative seizure parameters, and potentially identify those most useful for acute clinical management and long-term outcome prediction (68).

## 4 Conclusions

As of the current state of knowledge, NSE cannot yet be adequately defined, and focusing on the duration dimension alone seems to be an oversimplified approach.

A multidimensional definition incorporating temporal criteria, electroclinical semiology, treatment response and etiology is conceptually more appropriate and comprehensive. At the same time, we do recognize that a simplified operational definition that supports appropriate and timely intervention is equally needed.

The systematization of neuromonitoring, in both research and clinical contexts, for newborns experiencing or at high risk of seizures represents an essential first step in characterizing neonatal seizures across multiple dimensions, such as duration, recurrence, discharge pattern and spread area, absence or presence of clinical correlate, distinct semiology and treatment response, while identifying etiologies, specific patterns and enabling the subsequent outcome implications assessment.

Future trial designs (69) for NSE will need to separately examine the categories of acute symptomatic seizures and neonatal-onset epilepsies, or even specific etiological subgroups, and differentiate term and preterm. In order to better characterize and categorize the main features of this condition in the different subgroups, it is essential that these trials are based on the use of multichannel EEG tracings with simultaneous video and polygraphy recordings, initiated promptly, prolonged for adequate duration, and reviewed by expert readers. A systematic assessment of outcomes must be included, to analyze the correlation with the diverse features of NSE—after adjusting for other potential contributors specific to each etiology group—and therefore to identify the most significant descriptors to include in a multidimensional definition of NSE.

Further progress will depend on expanded access to continuous EEG monitoring, integration of new technologies in support to multichannel conventional EEG, implementation of exhaustive basic research studies, precision medicine tools, and implementation of standardized internationally accepted protocols across NICUs.

This approach would help build a fundamental base of knowledge upon which to develop a definition of NSE.

## Group members of the Neonatal Seizures and Epilepsy Committee of the Italian League Against Epilepsy

Elisabetta Cesaroni, Paola De Liso, Gaetano Cantalupo, Elisabetta Amadori, Luca Bartolini, Anna Cavalli, Lucrezia De Cosmo, Robertino Dilena, Valentina Gentile, Silvia Lori, Massimo Mastrangelo, Elena Pavlidis, Francesco Pisani, Jacopo Proietti, Federico Raviglione.
