# Supplementary material for: Neonatal status epilepticus: critical issues in clinical practice
Source: Front Neurol. 2026 Jul 16;17:1855017. doi: 10.3389/fneur.2026.1855017 (PMC13422202; doi:10.3389/fneur.2026.1855017)
Supplement: Supplementary file 1 [file Supplementary_file_1.docx]

| **DIMENSION** | **WHAT WE KNOW** | **CURRENT GAPS AND LIMITATIONS** | **FUTURE DIRECTIONS** | **REFERENCES** |
| --- | --- | --- | --- | --- |
| **Neonatal Brain** | Lower seizure threshold; incomplete myelination; possible resistance to seizure-induced injury | Age-specific injury mechanisms; effects of modified preterm exposome | Basic and translational research studies; exposome studies across gestational ages | 7-13, 15 |
| **Time/Duration** | Adult-derived T1 and T2 thresholds not appliable | NSE cut-off for a single  seizure and for the sum of multiple seizures within a given interval; NSE termination criteria; nature and impact of BRDs | Multicenter EEG-based studies to define T1/T2 | 1-5; 16-20; 24-28 |
| **Semiology** | ILAE 2021 classification (video-EEG mandatory); often electrographic-only; both electrographic and electroclinical seizures have influence on the outcome | Impact on outcome of specific semiology types; role of discharge features (frequency, amplitude, morphology, localization and spread) | Systematic semiology characterization based on video-EEG-polygraphy; full montage EEG in research settings; quantitative analysis of ictal EEG | 6; 33 |
| **Etiology** | Primary outcome determinant; acute symptomatic seizures and neonatal-onset epilepsies represent distinct categories; clinical context and semiology often guide the  etiology identification | Independent seizure contribution to neurological deterioration after adjusting for etiology contribution | Age-stratified and etiology-stratified trial designs (term vs. preterm; acute symptomatic seizures vs. neonatal onset epilepsies; dedicated studies in distinct etiological subgroups) | 37-38; 41-42 |
| **Outcome** | Prolonged and repeated seizures contribute to worse prognosis; established prognostic role of background EEG features | Meaningful NSE descriptors in terms of correlation with neurodevelopment | Rigorous assessment of outcomes included in NSE studies | 33; 43-44; 47-51 |
| **Treatment** | Timely treatment of NS  results in better response; PB remains first-line in most cases; targeted treatment emerging | Definition of therapeutic response, when to start and when to stop treatment | Precision medicine pathways; neuroprotection beyond hypothermia; ASM neurotoxicity trials | 21; 54; 56-59; |
| **Monitoring** | cEEG is the gold standard for seizure diagnosis (ILAE/ACNS) | Limited access to cEEG; selection bias represented by undetected seizures in non-monitored patients | Expanded cEEG access; AI-assisted seizure detection; standardized NICU protocols | 63-65 |

**Table 1: Summary.**
